# Editorial: Machine learning in computer-aided drug design

**DOI:** 10.3389/fmolb.2025.1568437

**Published:** 2025-02-25

**Authors:** Ognjen Perišić, Cigdem Sevim Bayrak, Mohamed Khaled Gunady

**Affiliations:** ^1^ Big Blue Genomics, Belgrade, Serbia; ^2^ Department of Genetics and Genomic Sciences, Icahn School of Medicine at Mount Sinai, New York, NY, United States; ^3^ Illumina, San Diego, CA, United States

**Keywords:** machine learning (ML), computer aided drug design, protein binding, hydration sites, antimicrobial activity, QSAR, antibody grouping, pharmacophores

The world population is growing. The United Nations estimates it will reach almost 10 billion by 2050 and 11 billion by the end of the century when the population curve will reach its maximum (United Nations Department of Economic and Social Affairs, 2018; Rosling et al., 2018). The increase in size will not come from the rise in the population of young people but from the rise in the population of adults and the elderly. This shift in demographics will highlight existing population health problems and bring new health-related challenges to the fore, with age-dependent cancer, neurodegenerative, cardiovascular, and chronic diseases continuing to be the dominant health issues. To address these problems, new drug development strategies will need to be devised that benefit both patients-by prolonging their comparable quality of life and reducing healthcare costs-and pharmaceutical developers by introducing more efficient, rapid, and cost-effective drug discovery and development techniques.

Drug discovery is a long and arduous process with a high risk of failure (Wong et al., 2019, Dowden and Munro, 2019). It takes more than a decade and more than a billion dollars to bring a single drug to market (which means that the total cost to pharma companies is even larger when accounting for failed drugs). The chance of a compound entering the preclinical stage and eventually being FDA-approved has been 1 in 20,000 to 30,000 over the last couple of decades (Yamaguchi et al., 2021). The cost and complexity of drug research have led major pharmaceutical companies to decrease their involvement in certain disease categories, such as cardiovascular and neurological diseases (Dowden and Munro, 2019), or to abandon early research and rely on acquisitions of smaller biotech companies that have drugs in preclinical or early clinical stages of development.

All these challenges have forced the pharmaceutical industry to accept *in silico* methods as a means of reducing costs and expediting development. Classical tools, such as molecular dynamics (MD), although offering a high level of accuracy and detailed insights into the behavior of proteins, are too expensive for high-throughput studies and are thus used only for evaluating targets and a small number of compounds. Those limitations opened a space for applying machine learning (ML) in drug development. While it has been used in academia for decades, with occasional excursions in the industry, ML came into the spotlight in recent years with the advancements in large language models (LLMs) and denoising diffusion probabilistic models and their use in computational structural biology. The successes of the AlphaFold and RosettaFold models, along with the subsequent Nobel Prize award to Demis Hassabis and John M. Jumper for protein fold prediction and to David Baker for computational protein design, led many to believe that the majority of structural biology and, relatedly, drug design problems would be easily solved. Those were high hopes because ML, although powerful, has limitations. One of the most significant limitations of ML models is their poor generalization outside of training space, making them strongly dependent on the compositions of the training set. An additional issue is, paradoxically, the simplicity with which it is now possible to implement ML models. Modern, advanced ML libraries (PyTorch and TensorFlow) enable the easy deployment of ML models, often without delving into the details of biological phenomena being analyzed. This can lead to a superficial understanding of the results obtained with an ML model. Furthermore, the “black-box” nature of ML models often creates challenges for their adaptation in medical applications, and drug discovery. To bridge the gap between computational power and complex biological systems, interpretable models are needed.

With all this in mind, we conceptualize this Research Topic with the idea of presenting research that utilizes ML protocols/architectures but offers a detailed and comprehensive interpretation of observed phenomena.

The first paper in this Research Topic, by Chen et al., deals with the detection of peptides that can bind major histocompatibility complex (MHC) class-I proteins. The authors designed two Convolutional Neural Network-based methods, ConvM and SpConvM, to tackle the binding prediction problem and conducted a thorough bioinformatics study of the results. They show that their method outperforms the current state-of-the-art, allele-specific method in prioritizing and identifying the most likely binding peptides.

Huang et al. addressed the detection of hydration sites in proteins and the prediction of water molecule positions using ML. This is an important issue in drug design as the analysis conducted prior indicates that the majority of ligand binding sites in protein-ligand structures contain at least one bridging water molecule at the interface. The authors’ two-component (scoring and sampling) model outperformed alternative approaches by a large margin.

The next paper also deals with peptide classification. Khabaz et al., developed a hierarchical machine-learning model for classifying peptides with antimicrobial activity against *S. aureus*. Their two-level model first classifies peptides into Anti-Microbial Peptides (AMPs) and non-AMPs. The second level then classifies AMPs as active and inactive against *S. aureus*. The model uses linguistic and physicochemical properties, which were selected through cross-validation-based feature selection to identify the most important features. The model can be used in drug discovery, peptide design, and functional annotation of peptides.

Faris et al., developed a method for discovering selective inhibitors against JAK1 and JAK3. The method uses QSAR models optimized with multiple linear regression and artificial neural networks (ANN). It enabled the identification of optimal compounds exhibiting both favorable affinity and stability during a 100 ns molecular dynamics trajectory. This approach, developed with the help of ANNs, has demonstrated its capability to predict biological activity and stability.

Chomicz et al., used clustering and machine learning protocols to develop a method for antibody grouping using clonotype, sequence, paratope prediction, structure prediction, and embedding information. The authors used advanced methods for fast sequence clustering and language models to cluster paratopes. For structure clustering, they applied an adaptation of AlphaFold2 to model antibodies and a fast greedy algorithm-based tool for similarity estimation. The last layer in their architecture is a self-supervised embedding-based language model. They use it to cluster antibody sequences in the latent space. Their results indicate that novel, ML-based methods offer no advantage over standard sequence-based tools for probe-based binder mining. However, they noticed that the advanced ML methods are useful for epitope binning. Thus the authors conclude that advanced methods are better suited for separating a given dataset, rather than to perform data-mining experiments.

Ahmadi et al. developed a machine-learning protocol that uses pharmacophore features to separate true binding ligands from decoys for four protein targets. They first used molecular dynamics simulation to generate pharmacophore feature sets from protein-ligand complex conformations. Then, they applied AI/ML algorithms to reduce the whole set of those features to a much smaller set. They showed that this protocol is effective for true binder prediction while remaining medicinal-chemistry friendly.

The papers published in this Research Topic focus on leveraging machine learning to analyze biological models, predict molecular behaviors, and aid in drug discovery. They incorporate ML into diverse applications, such as peptide-MHC binding prediction, protein-ligand interaction prediction, antimicrobial peptide classification, and antibody clustering. While also demonstrating how these protocols can identify both small-molecule and antibody binders, providing meaningful biological insights.
